# Evidence-based recommendations for gastrointestinal cancers during the COVID-19 pandemic by the Brazilian Gastrointestinal Tumours Group

**DOI:** 10.3332/ecancer.2020.1048

**Published:** 2020-05-22

**Authors:** Rachel P Riechelmann, Renata D’Alpino Peixoto, Gustavo Dos Santos Fernandes, Rui F Weschenfelder, Gabriel Prolla, Duílio Rocha Filho, Aline Chaves Andrade, Marcela Crosara, Juliana Florinda M Rego, Rene C Gansl, Felipe Coimbra, Samuel Aguiar, Elisângela Carvalho, Paulo M Hoff, Anelisa K Coutinho

**Affiliations:** 1 AC Camargo Cancer Center, Sao Paulo, SP 01509-010, Brazil; 2 Hospital Oswaldo Cruz, Sao Paulo, SP 01323-020, Brazil; 3 Hospital Sírio Libanês, Distrito Federal, DF 70200-730, Brazil; 4 Hospital Moinhos de Vento, Porto Alegre, RS 90035-000, Brazil; 5 Centro de Oncologia Hospital São Lucas da PUCRS, Porto Alegre, RS 90610-000, Brazil; 6 Hospital Universitário Walter Cantídio, Foratleza, CE 60430-372, Brazil; 7 Grupo Oncoclínicas, Belo Horizonte, MG 30360-680, Brazil; 8 Rede D’Or, DF 70390-140, Brazil; 9 Hospital Universitário Onofre Lopes, Natal, RN 59012-300, Brazil; 10 Hospital Israelita Albert Einstein, Sao Paulo, SP 05652-900, Brazil; 11 Hospital São Rafael, Salvador, 41253-900, Brazil; 12 Instituto do Cancer do Estado de São Paulo, Faculdade de Medicina da Universidade de São Paulo, Sao Paulo, SP 01246-000, Brazil; 13 Clinica AMO, Assistência Multidisciplinar em Oncologia, Salvador, BA 41950-640, Brazil

**Keywords:** COVID-19, cancer, gastrointestinal tumours, pandemic, coronavirus

## Abstract

**Purpose:**

As of 2020, the world is facing the great challenge of the COVID-19 (Coronavirus disease 2019) pandemic, caused by the SARS-CoV-2 virus. While the overall mortality is low, the virus is highly virulent and may infect millions of people worldwide. This will consequently burden health systems, particularly by those individuals considered to be at high risk of severe complications from COVID-19. Such risk factors include advanced age, cardiovascular and pulmonary diseases, diabetes and cancer. However, few data on the outcomes of cancer patients infected by SARS CoV-2 exist. Therefore, there is a lack of guidance on how to manage cancer patients during the pandemic. We sought to propose specific recommendations about the management of patients with gastrointestinal malignancies.

**Methods:**

The Brazilian Gastrointestinal Tumours Group board of directors and members sought up-to-date scientific literature on each tumour type and discussed all recommendations by virtual meetings to provide evidence-based—and sometimes, expert opinion—recommendation statements. Our objectives were to recommend evidence-based approaches to both treat and minimise the risk of COVID-19 for cancer patients, and simultaneously propose how to decrease the use of hospital resources at a time these resources need to be available to treat COVID-19 patients.

**Results:**

Overall and tumour-specific recommendations were made by stage (including surgical, locoregional, radiotherapy, systemic treatments and follow-up strategies) for the most common gastrointestinal malignancies: esophagus, gastric, pancreas, bile duct, hepatocellular, colorectal, anal cancer and neuroendocrine tumours.

**Conclusions:**

Our recommendations emphasise the importance of treating cancer patients, using the best evidence available, while simultaneously taking into consideration the world-wide health resource hyperutilisation to treat non-cancer COVID-19 patients.

## Introduction

The year of 2020 will be forever remembered as the year of the COVID-19 (Coronavirus disease 2019) pandemic, caused by a new coronavirus named SARS-CoV-2. The new coronavirus surged in the Chinese province of Hubei in early December 2019 and as of May 5th 2020 more than 3,500,000 people around the globe have been infected and nearly 250,000 have lost their lives throughout 208 countries and territories. Individuals mostly at risk of severe complications are those with cardiovascular and/or pulmonary diseases, diabetes mellitus and the elderly. Because of an inherent immunosuppressive condition, cancer patients are considered to be particularly prone to developing serious complications from COVID-19. In the Chinese experience from Wuhan, 18 patients out of 1,590 infected by SARS-CoV-2 had solid tumours and, in comparison with non-cancer patients, they experienced more serious events (intensive care admission with or without mechanical ventilation or death): 39% versus 8%. Cancer patients who received chemotherapy or underwent surgery in the previous month were at an even higher risk of severe events: three (75%) out of four versus six (43%) out of 14 patients [1].

Because, it is estimated that approximately 10% of patients with COVID-19 will need hospitalisation, an overload, or even a collapse, of health systems is expected. Such a burden will certainly impact on the treatments of millions of people with various different diseases, including cancer. Since cancer is a severe disease, patients with malignancies need to be promptly treated for their tumours. Hence, health institutions around the world will have to balance how they will manage medical care for COVID-19 and cancer patients (plus all other severe medical conditions). Foreseeing that hospitals and medical staff will be burdened by the pandemic, international oncology societies have sought to provide general guidelines about systemic and surgical cancer treatments with the aim to decrease health resource utilisation, such as medical visits, exams and delay surgical procedures [2–4]. The Society of Surgical Oncology, for example, recommends that all cancer patients who are candidates for oncological surgeries be tested for COVID-19 prior to surgery [5], given that asymptomatic patients with SARS-CoV-2 undergoing elective surgery experience significant worse clinical outcomes, including higher mortality [6]. The American Society of Clinical Oncology website provides information for patients and medical staff about infection prevention as well as overall tips about reducing the number of hospital visits for patients (e.g., if possible, choose oral rather than intravenous cancer-directed therapies) (https://www.asco.org/asco-coronavirus-information). The European Society of Medical Oncology (ESMO) website delivers sessions on frequent questions and answers about COVID-19, encourages telemedicine and also offers guidance on how to reduce hospital visits and information for patients (https://www.esmo.org/covid-19-and-cancer?hit=ehp).

Yet, specific and detailed treatment recommendations by tumour types and related treatments have not been widely available. Ahead of other oncology societies, ESMO has issued more specific recommendations for some gastrointestinal cancers (colorectal, esophagus and pancreatic) and propose treatments based on priority levels; however, direct treatment recommendations for each tumour stage are not provided [7]. Therefore, we think it is useful to propose practical and precise recommendations on how to manage patients with gastrointestinal malignancies during the COVID-19 pandemic.

## Methods

This initiative was developed by the board of directors of the Brazilian Gastrointestinal Tumours Group (GTG), a nonprofit organisation founded in 2011 and devoted to medical education and research in gastrointestinal cancers. We sought up-to-date literature to provide all recommendations for each clinical and/or pathological stage of the most common gastrointestinal cancers (esophageal, gastric, pancreas, bile duct, hepatocellular, neuroendocrine, colorectal and anal cancer). We had virtual meetings to discuss all the topics, reaching consensus on all statements. All recommendations made here were based on the best available scientific evidence, such as randomised clinical trials, meta-analyses and large cohort studies. However, in some scenarios where solid evidence was lacking, we used common sense to provide an expert opinion recommendation (marked in the text as ‘EOR’). All the recommendations proposed in this manuscript, besides being evidence-based, aimed at the following objectives: (1) to prioritise curative-intent cancer treatments during the pandemic; (2) to support the treatment of aggressive tumours when effective therapies are available; (3) to decrease the number of or delay oncological non-priority surgeries; (4) to decrease hospital visits (e.g., substitute intravenous for similarly effective oral drugs; propose when and to whom treatments delays/interruption/watchful waiting can be offered); (5) to minimise anticancer therapy-related immunosuppression in specific high-risk groups (here defined as: elderly, comorbid illnesses [especially diabetes, cardiovascular and/or pulmonary concurrent diseases], fragility, ECOG 2 or higher).

All recommendations are made for scenarios where services still have the capacity to treat cancer patients but are expected to destiny some of their space, beds and staff to manage COVID-19 patients.

## Overall recommendations

- Social distancing mandates that every in-person interaction between patients and the health care system be scrutinised and only essential physical contacts between patients and health care professionals occur to diminish the risk of viral exposure to patients. Thus, minimise blood tests, scans and routine tests. Telephone and telemedicine visits should replace routine face-to-face clinic visits whenever possible.
- Whenever COVID-19 is clinically suspected or confirmed, systemic treatments should be suspended, and surgery should be postponed unless an urgent procedure is necessary. (EOR)
- Whenever surgery is indicated, SARS-CoV-2 testing should be considered [8, 9].
- There are insufficient data to recommend in favour or against an open versus minimally invasive approach. Proven benefits of minimally invasive surgeries of reduced length of stay and complications should be considered individually [10]. Nevertheless, whenever minimally invasive surgeries are indicated, the use of devices to filter released CO_2_ for aerosolised particles or techniques to treat the intra-abdominal gas whenever it should be emptied, are strongly advised [11].
- Central venous catheter flushing intervals should be increased to every 60 (younger and fit patients) or to every 90 (older, frail patients with multiple comorbidities) days. (EOR)
- For early stage (cT1/2 cN0) colorectal, biliary, hepatocellular, esophagus and gastric tumours, where neoadjuvant treatment is not standard, consider deferring surgical resection to up to 8 weeks. If delays beyond 8 weeks are expected, repeat staging exams. (EOR)
- Radiation schedules should be hypofractionated, whenever possible.
- Follow-up imaging and appointments should be reserved for those with symptoms suggestive of disease relapse. Asymptomatic patients not on active treatment should avoid imaging and follow up appointments, delaying tumour markers and colonoscopies, for example, for until the pandemic is over. (EOR) In such cases, if possible, telemedicine or telephone consultation is indicated.
- DYPD screening is indicated whenever possible, before the use of fluoropyrimidines [12].
- Adjuvant treatment for colon [13] and other gastrointestinal tumours, when recommended, should start in 4 to 8 weeks after primary tumour resection. Monitoring blood counts at every cycle can be done by telemedicine if patients are asymptomatic.
- Infusional 5FU should be substituted for capecitabine in the following regimens: FOLFOX, cisplatin and 5FU, monotherapy or when combined with radiotherapy [14–16]. Exceptions are patients with severe renal dysfunction (creatinine clearance ≤ 30 ml/minute); in patients with moderate (30 to 50 ml/minute) renal dysfunction when upfront dose reduction of 25% is recommended.
- In curative-intent treatments, we encourage to maintain dose-intensity with the use of colony-stimulating growth factor (CSGF), if needed. (EOR)
- In the metastatic setting, consider dose-reduce chemotherapy instead of adding CSGF, if the latter requires more hospital visits. (EOR)
- In the metastatic setting, omit *bolus* 5FU in FOLFOX or FOLFIRI regimens to minimise toxicity. (EOR)
- Whenever possible, chemotherapy holidays may be considered in patients with low-volume metastatic disease, who are responding or experiencing tumour stabilisation and when there is no major risk of complications for site-specific progression (e.g., peritoneum, biliary obstruction). If maintenance is considered to be beneficial instead of chemoholidays (e.g., more aggressive disease), prefer capecitabine alone, without bevacizumab.
- Standard second or further lines of anticancer therapies should be recommended for ECOG 0 or 1 patients. Preferably, when there is clinically relevant overall survival gain demonstrated by randomised phase III trials (e.g., second-line for colorectal cancer) [2].
- Anti-PD1 immune check point inhibitors are recommended in second or further lines of treatment for *all* gastrointestinal malignancies with microsatellite instability, regardless of the diagnostic method [17].
- For those in which immunotherapy monotherapy is indicated, we recommend the 6 weeks schedule with pembrolizumab [18].
- Multidisciplinary team discussions (MDT) by web conferencing systems are highly encouraged. We think MDT are key to help with decisions about risks and benefits of cancer-directed therapies during the COVID-19 pandemic.
- In all cases, clinical individual judgment is advised and decisions should be shared with patients. Additionally, the anticipated survival benefit for each patient versus the risks of exposure to the virus should be discussed with patients, taking into consideration the individual’s comorbidities and degree of frailty, as well as caregivers and family members at home.
- Clinical trial enrolment:
  - Patients who are candidates for clinical trials should be encouraged to enrol in the following situations: studies testing orphan drug indications, experimental treatments where benefits are very likely to outweigh the risks (e.g., immunotherapy combo of ipilimumab and nivolumab for microsatellite unstable metastatic colorectal cancer (CheckMate 8HW – NCT 04008030) or rare tumours. However, institutions and principal investigators should discuss and align with sponsors and Institutional Research Ethical Boards about how to minimise hospital visits (e.g., all lab and image tests performed in one single day), implement telemedicine in certain moments of trial conduction (lab checks for fit patients who are tolerating well the trial therapy, for example), extend intervals between hospital visits, if possible.
  - For patients already on trial, treatment should continue based on clinical judgement that should balance tolerance versus benefit. The same principles cited above to decrease hospital visits should be sought.

## Recommendations by tumour types

### Esophagus

#### Early stage

- cTis, cT1a/b cN0:

- cT1a lesions amenable to endoscopic resection may preferentially undergo endoscopic management [19]. pT1b adenocarcinomas may also be considered for endoscopic resection when there is no evidence of lymph node metastases, or lymphovascular invasion and/or poor differentiation, or in elderly and/or high-risk patients. (EOR)
- If endoscopic treatment is not possible, consider deferring the intervention up to 8 weeks in younger and fit patients or up to 12 weeks in older, frail patients (see Overall Recommendation).

- cT2-T4 and/or clinically lymph-node positive (cN+):

- Staging with 18-FDG-PET should be performed, because it detects up to 20% more distant metastasis in comparison to conventional computerised tomography (CT) [20].
- Staging laparoscopy is not recommended, to avoid aerosol exposure of staff involved in the procedure [8].
- Perioperative chemotherapy with FLOT with CSGF is preferred over chemoradiation in gastroesophageal junction and Siewert III adenocarcinoma [21] (and EOR). If the patient is responding, consider performing all cycles of programmed chemotherapy preoperatively. (EOR)
- In Siewert I and II subtypes, chemoradiation following the CROSS regimen is advised [22].
- For patients who did not receive neoadjuvant chemotherapy, adjuvant treatment should be prescribed whenever indicated (pT3 or higher and/or pN+) and may be postponed up to 12 weeks after surgery [23].
- Adjuvant radiation therapy alone should be avoided, except for R1 resection.
- Squamous cell carcinoma should be treated with neoadjuvant chemoradiation. The association of carboplatin, paclitaxel and radiotherapy (CROSS trial protocol) should be preferred due to better toxicity profile. Radiation dose should be limited to 41.4 Gy to reduce the number of visits to the clinic [22].
- Patients with squamous cell carcinoma and complete clinical response to chemoradiation could avoid surgery [24].
- In locally advanced/unresectable esophageal cancer, chemotherapy alone may be considered, instead of chemoradiotherapy. (EOR) When definitive chemoradiation is indicated, weekly carboplatin and paclitaxel should be considered and radiation dose should be increased to 50 Gy. (EOR)
- Patients with obstructive symptoms or haemorrhage may be treated with endoscopic measures and proceed to surgery if these measures fail [19]. If radiotherapy is necessary, ultra-hypofractioned schemes are recommended.

#### Metastatic disease

- Doublets (Oxaliplatin + capecitabine or cisplatin + capecitabine) are preferred over triplets and should be prescribed until progression if tolerance allows.
- Consider up-front or on-treatment dose-reductions up to 60% according to risk-benefit assessment. (EOR)
- Earlier imaging (every 8 weeks) is recommended. Consider discontinuation of systemic treatment when results are not favourable.
- For patients with ECOG 0 or 1, second line pembrolizumab is indicated in adenocarcinoma or squamous cell tumours with CPS >= 10 [25].
- For patients who are not candidates for systemic treatment but require management of local symptoms, consider palliative radiotherapy (single fraction or hypofractionated schemes) or stent.

### Gastric cancer

#### Early stage

- cT1a lesions amenable to endoscopic resection should preferentially undergo endoscopic management [5]. Given the concerns for aerosolisation with endoscopic procedures, ensuring patients are COVID-19 negative and/or postponing the procedure should be considered (see Overall Recommendations).
- cT1b cN0 cancers should be resected, although surgery could be temporarily postponed up to 8 weeks after MDT. (see Overall Recommendations)
- cT2 cN0 cancers could either be considered for upfront surgery or neoadjuvant therapy. (EOR)
- cT3 or higher and/or cN+ should be treated with neoadjuvant systemic therapy [19, 26, 27]. Patients should initiate chemotherapy without delays whenever possible. Those who are already on neoadjuvant chemotherapy and responding to it with reasonable tolerance should complete all perioperative treatment so that surgery could be postponed. (EOR)
- Diagnostic laparoscopy to rule out metastatic disease should be avoided whenever hospital resources or space is critical. However, when not previously done, consider diagnostic laparoscopy after neoadjuvant therapy. (EOR)
- Adjuvant treatment should be prescribed whenever indicated (pT3 or higher and/or pN+) and may be postponed up to 8 weeks after surgery [23].
- Adjuvant radiation therapy should be avoided, except for R1 resection and/or D0 gastrectomy.
- For patients with MSI-H tumours and localised disease, surgery should be the only treatment; no chemotherapy or radiation is indicated [28].

#### Metastatic

- Triplets, such as DCF and FLOT, should be avoided in the metastatic setting to minimise the risk of hospital admission.
- Metastasectomies should be avoided [19, 26, 27].
- Primary tumour complications, such as bleeding and obstructions, should be discussed in MDT and managed according to local resources.

### Pancreatic cancer

#### Resectable and borderline resectable tumours

- Consider staging with 18-FDG PET-CT to avoid surgery in subclinical stage IV patients [29].
- Consider neoadjuvant therapy for all patients, including upfront resectable (e.g., cT2 cN0) patients who are fit and obvious candidates for adjuvant treatment [30, 31]. Histological diagnosis is mandatory before starting chemotherapy.
- Neoadjuvant treatment is strongly recommended for patients with borderline tumours based on vascular or biological criteria (i.e., Ca19.9 > 500 U/ml; enlarged regional lymph nodes). Options include hyporfactionated precision radiation (25–35 Gy/5 fractions) or chemoradiation (36 Gy/15 fractions) with concurrent capecitabine (modified from PREOPANC trial) [31], or modified FOLFIRINOX [32] with GCSF support for fit patients.
- Patients fit for adjuvant modified FOLFIRINOX should not have their treatment postponed. Those without clinical conditions (elderly, frail, multiple comorbid illnesses) for FOLFIRINOX should receive adjuvant gemcitabine [33, 34] or capecitabine monotherapy or even no adjuvant treatment. (EOR)

#### Metastatic

- Systemic treatment only for ECOG 0–2 patients. ECOG 3–4 patients are candidates for best supportive care only.
- Modified FOLFIRINOX or Gemcitabine and nab-paclitaxel are recommended for first line [35, 36]. Gemcitabine and nab-paclitaxel require less hospital visits and could avoid surgery to implant the totally implantable catheters.
- Reduction of chemotherapy intensity (PANOPTIMOX trial strategy) is strongly recommended for patients whose tumour are responding/stable on FOLFIRINOX [37].
- Patients with known pathogenic or likely pathogenic BRCA germline mutations may receive cisplatin and gemcitabine as a first or second-line therapy [38]. (and EOR). Maintenance with olaparib can be offered to fit patients who are responding to a platinum-based regimen.

### Bile duct cancers

#### Early stage

- Patients with resected biliary tract cancers should be offered adjuvant capecitabine chemotherapy for a duration of 6 months [39].
- For early stage tumours (N0), with free margins in patients who are 70 years or older or those with risk factors for severe COVID-19, consider not offering adjuvant chemotherapy. (EOR)

#### Metastatic

- Systemic first-line chemotherapy with cisplatin and gemcitabine should be administered in ECOG 0–2 patients [40].

### Hepatocellular carcinoma

#### Localised disease

- Barcelona Clinic Liver Cancer (BCLC) staging 0 and A patients: organ transplantation should not be delayed [41].
- 18-FDG PET-CT should be performed, if available, to avoid surgery in subclinical metastatic disease [42].
- BCLC stages A and B patients should be evaluated for locoregional therapy [19].
  - BCLC A patients should be considered for minimally invasive treatments: prefer radiofrequency ablation or stereotactic radiotherapy over surgery to avoid major complications. (EOR)
  - When chemoembolisation is the chosen option, try to diminish hospitalisation time by monitoring liver function on an outpatient basis. (EOR)

#### Advanced disease

- BCLC stage B patients who are not candidates for locoregional therapy and BCLC stage C patients should be offered systemic therapy. Select patients who are most likely to benefit based on performance status, Child-Pugh score and comorbidities.
  - Atezolizumab and Bevacizumab should be considered, when available, as the first line of choice, for fit patients, based on performance status, Child-Pugh score and comorbidities [43]. If this option is not available, sorafenib or lenvatinib are the recommended first-line treatments [44, 45].
- Consider omitting radiology response assessment and continue to clinical progression according to tolerance and tumour marker alpha-fetoprotein. (EOR)
- Regorafenib should be used in second line for ECOG 0/1 patients who experienced good tolerance to first line multi-kinase inhibitor [46]. Regorafenib should preferably be initiated in a dose-escalation schedule, (EOR) resembling the ReDos scheme administered for metastatic colorectal cancer [47].
- Consider ramucirumab for second line if alpha fetoprotein is > 400 ng/ml in patients who are intolerant to multi-kinase inhibitors [48].
- Avoid any systemic treatment for Child-Pugh B patients [49].

### Neuroendocrine tumours

#### Well-differentiated neuroendocrine tumours (NET)

Localised NET:

- Surgical procedures in patients with NET should follow standard recommendations [50]. Overall, symptomatic tumours should be removed and small non-functioning Grade 1 tumours could have surgery deferred by up to 90 days. (EOR)
- Liver transplantation, because of its experimental basis and induced immunosuppression, should not be considered during the pandemic. (EOR)

Metastatic NET:

- Resectable G1 gastroenteropancreatic (GEP) NETs with liver-only metastases should have their surgery deferred based on biological behaviour. For non-functioning tumours, watchful waiting is recommended, with follow up images performed at every 4 to 6 months [50].
- For Grade 2 or progressing GEP NET, if an octreoscan or Galium-68 DOTATATE PET-CT demonstrate somatostatin receptor expression, long acting somatostatin analogues are the preferred first line option [50].
- Functioning NETs should always receive somatostatin analogues.
- In cases of Grade 3 NET which present with an indolent course, somatostatin analogues can be used, with radiological controls performed every 3 months. (EOR)
- For patients with disease progression following somatostatin analogues, we recommend second-line therapy with:
  - For midgut: peptide-receptor radionuclide therapy (PRRT), if significant somatostatin receptor expression is documented in an octreoscan or Galium-68 DOTATATE PET-CT. If functioning images are negative, we suggest bland hepatic embolisation for liver-predominant disease [50].
  - For pancreatic NET: PRRT or sunitinib are recommended for more indolent tumours [50, 51].
  - For more aggressive NETs, we recommend capecitabine and temozolamide [52].
  - For liver-predominant disease, hepatic embolisation can be used and repeated to treat progressive disease and/or refractory carcinoid syndrome, if adequate liver function is preserved [50].
  - We suggest avoiding the use of everolimus during the pandemic because of the risk of severe and opportunistic infections [53].

Poorly-differentiated neuroendocrine carcinomas (NEC):

- In patients with small localised NEC, surgery should be performed without delay. For cN+ or bulky NEC, start with systemic chemotherapy (see metastatic disease). Consider staging patients with 18-FDG PET-CT scan prior to surgery to rule out metastatic spread [50].
- For localised rectal or esophageal NEC, chemoradiation with cisplatin and etoposide should be initiated [50]. For patients who undergo R0 resection and with good performance status, consider 4 to 6 cycles of adjuvant cisplatin and irinotecan or etoposide. (EOR)
- Metastatic disease should be treated with cisplatin-based regimens.
- Evaluate MSI status in tumour tissues if immunotherapy is available for metastatic disease.

### Localised rectal adenocarcinoma

- We have adapted our recommendations from the recently published ESMO recommendations on rectal cancer [54].
- High rectal tumours (intraperitoneal location) follow the same recommendations for colon cancer.
- For cT1-2 or cT3a N0 (middle and low with sphincter preservation), with clear circumferential margins (CRM), proceed with total mesorectum excision (TME) without pre-operative radiotherapy.
- For low-risk T1, transanal endoscopic microsurgery (TEMS) can be considered.
- For cT3b/c or cN+ (middle or low rectum) with clear circumferencial margins, we recommend short course radiation therapy with delayed surgery. (EOR) If major response is needed for sphincter preservation, we favor long-course chemoradiation. (EOR) Importantly, short course radiotherapy, while leading to less hospital visits, is associated with more G3 or 4 treatment-related adverse events (mainly gastrointestinal) when compared with long-course radiation [58].
- For cT4, or threatened/involved CRM, or lateral pelvic lymph nodes, or suspected cN2/bulky lymph nodes involvement, we favour total neoadjuvant therapy with long-course chemoradiation with capecitabine followed by four to six cycles of chemotherapy (mFOLFOX6) or short-course radiotherapy followed by four to six cycles of chemotherapy (mFOLFOX6). CAPOX can substitute for mFOLFOX6 in younger and/or fit patients with normal renal function and without an ileostomy [55–57]. Chemotherapy followed by chemoradiation is also an adequate option in total neoadjuvant therapy for rectal cancer. Institutions should start with the promptest modality. (EOR)
- Patients in complete clinical remission after completion of neoadjuvant therapy can have surgery delayed and might be offered a watch-and-wait approach for low rectal tumours. Patients whose tumours have been clearly downstaged can have surgery delayed or avoided if a further follow-up demonstrates complete clinical remission [59, 60].
- Postoperative chemoradiation should be considered in patients who did not have pre-operative radiation and with adverse pathological features, such as: positive circumferential margin, incomplete mesorectal resection, extranodal deposits, nodal deposit with extracapsular spread close to the mesorectal fascia, extensive extramural vascular invasion/perineural invasion close to the mesorectal fascia, and/or pN2 low tumours within 4 cm of the anal verge [61].
- The decision regarding adjuvant chemotherapy for patients who were operated upfront will be the same as discussed in colon cancer section.
- In elderly and/or frail patients or those not fit for chemotherapy or standard chemoradiation, we recommend short course radiation with a delay of 8 to 12 weeks to surgery. (EOR)
- Diverting stoma is highly recommended for all extraperitoneal colorectal anastomoses during the pandemic, as it minimises the severity of complications. (EOR)

### Colon cancer

#### Early stages

- Depending on the pandemic phase and availability of hospital beds, it is acceptable to start neoadjuvant chemotherapy [62] if surgery is expected to be delayed by more than 8 weeks.
- Adjuvant chemotherapy for stage II colon cancer is recommended in patients with the only following high-risk features: less than 12 examined lymph nodes and/or pT4 and/or perforated tumours and microsatellite stability (MSS) or proficient mismatch repair (pMMR); other risk factors, such as poor cell differentiation or vascular invasion, if isolated, should not solely guide adjuvant treatment in stage II colon cancer. (EOR)
- Adjuvant therapy is not indicated for patients with stage II microsatellite- high (MSI-H) or deficient mismatch repair (dMMR) tumours.
- Preclude adjuvant chemotherapy for patients who are 70 or more years old. (EOR)

#### Stage III

- Patients with low risk (pT3N1) and pT3N2 tumours should receive CAPOX for 3 months [63].
- Patients not fit for oxaliplatin, either capecitabine or LV5FU2 (de Gramont) infusion, are acceptable adjuvant regimens for a 6-month duration.
- Those with pT4 tumours should be treated with CAPOX for 6 months [63]; if G2 or higher peripheral neuropathy, discontinue oxaliplatin and maintain capecitabine.
- For 70 years or older, consider capecitabine for 6 months

#### Metastatic colon:

-Resectable metastases/borderline:

- For up-front or borderline resectable disease consider chemotherapy with CAPOX for up to eight cycles. The timing of surgery can be flexible but evaluate response by radiological images every 2 to 3 months. (EOR)
- 18-FDG PET-CT is recommended for staging prior to first chemotherapy, with the aim to detect subclinical metastatic disease [64].

Incurable metastatic disease:

- In first line, prefer capecitabine + bevacizumab for low volume of disease and/or age 70 years or older [65, 66].
- Doublets with or without monoclonal antibodies in moderate/high volume of disease, following the standard options, but always taking into consideration patient’s comorbidities, RAS/BRAFv600E mutation status, MSI-H status and sidedness.
- When a plateau of response is achieved, consider maintenance with capecitabine or chemotherapy holidays, with images performed every 12 weeks [67].
- Avoid triplet chemotherapy regimens (FOLFOXIRI) with or without monoclonal antibodies [68], except for patients with high burden of disease and young/fit patients and/or with BRAF V600E mutated tumours [69]. (and EOR)
- Synchronic metastatic colon or rectal tumours should be initially treated with systemic treatment. (EOR) These cases should be discussed and their management decided in MDT.
- Regarding second-line treatments, follow the standard rationale, taking in consideration clinical performance status, first line regimens, responses, volume of disease and molecular markers.
- For patients who have failed standard chemotherapy and monoclonal antibodies, regorafenib or trifluridine/tipiracil are indicated. When regorafenib is used, start with a dose-escalation schedule as studied in the ReDOS trial [47].
- In later lines, for patients whose cancers have either HER2 amplification, BRAF V600E mutations, or are RAS/RAF wild-type, need treatment and are refractory to 5-FU/capecitabine, consider switching to targeted therapies alone, without concurrent use of cytotoxic agents [70].
- Weekly cetuximab should be avoided. Instead, use every-other-week anti-epidermal growth factor receptor (EGFR) dosing [70].

### Squamous cell carcinoma of the anus

- Patients with cT1N0M0 tumours can be treated with local excision if a 1cm margin can be achieved [71].
- Patients with cT2-4N0-1M0 should receive standard chemoradiation with mitomicyn or cisplatin combined with capecitabine 825 mg/m^2 ^BID (ref) on radiation treatment days [72].
- Patients with cT2N0M0 and 70 years or older and/or with multiple comorbid illness can be treated with radiation alone with or without capecitabine. (EOR)
- For patients with locally relapsed/persistent/progressed tumours, salvage anorectum amputation should be performed; if available, an 18-FDG-PET-CT should be performed to avoid unnecessary radical surgery in the context of metastatic disease [73].
- The first line recommended regimen for metastatic disease is carboplatin and paclitaxel, because it offers less serious adverse events over conventional cisplatin and infusional 5-FU [74].
- The modified DCF regimen can be used in selected ECOG 0 and 1 fit patients with bulky visceral metastatic disease [75].
- In second-line, immune check point inhibitors should be considered for ECOG PS 0-1 [76, 77].
- Particularities in anal cancer in patients with HIV infection:
- Localised non-metastatic disease: initiate full chemoradiation in patients with CD4 count higher than 240 cell/mm^3^; cisplatin or mitomycin offer similar outcomes [78]. It is acceptable to dose reduce chemotherapy in patients with CD4 count of less than 240 cell/mm^3^ although there is no standard schedule. (EOR)
- Anal cancer in HIV-infected patients take longer time to achieve clinical complete response, with nearly two thirds presenting complete response after 6 months. Therefore, we recommend waiting longer (at least one year for those without local progression) before recommending salvage anorectum amputation in these patients [78].
- Treatment for metastatic disease should follow the same principles for non-HIV-infected patients.

## Conclusions

Given the unprecedented moment health care systems and oncologists are facing worldwide, the recommendations proposed here by the Brazilian Gastrointestinal Tumours Group provide a balance of how to best manage gastrointestinal tumours in the face of the SARS CoV2 pandemic, taking into consideration the limited health resources available in many countries. We think that a practical and detailed set of recommendations may help general oncologists to better (and safely) treat their patients with gastrointestinal cancers.

## Conflicts of interest

**Rachel Riechelmann:** honoraria and consultancy for Bayer, Astra Zeneca, Roche, Novartis, Ipsen, Servier. Research grants: Bayer, Amgen, Roche, Libbs.

**Renata D’Alpino Peixoto:** honoraria and consultancy for Bayer, Roche, Lilly, Novartis, Ipsen, Amgen, Merck, EMS.

**Gustavo Fernandes:** honoraria and consultancy for Bayer, Roche, Novartis, MSD. Research : BMS, Roche, MSD.

**Aline Chaves Andrade :** honoraria and consultancy for Bayer, Novartis, Astra Zeneca, Ipsen, Amgen.

**Rui F Weschenfelder:** honoraria and consultancy for Amgen, Bayer, Ipsen, Lilly, Merck, MSD, Sanofi, Roche.

**Juliana Florinda M Rêgo:** honoraria and consultancy for Bayer, Novartis, Ipsen.

**Gabriel Prolla:** none.

**Duilio Rocha Filho:** honoraria and consultancy for Bayer, Merck Serono, Novartis, Ipsen, Servier

**Marcela Crosara:** honoraria and consultancy for Lilly, MSD and Roche.

**Rene C Gansl:** honoraria for Novartis and Ipsen

**Felipe Coimbra:** none.

**Samuel Aguiar Jr:** none.

**Elisangela Carvalho:** none.

**Paulo M Hoff:** none.

**Anelisa K Coutinho:** honoraria and consultancy for Amgen, Bayer, Ipsen, Lilly, Merck, MSD, Roche, Sanofi, Servier.

## Funding

This work received no external funding.
